# Hyaluronan-Based Glioblastoma Tumor Constructs Maintain Patient Tumor Drug Responses and Genomic Parity

**DOI:** 10.3390/mi17030276

**Published:** 2026-02-24

**Authors:** Hemamylammal Sivakumar, Steven D. Forsythe, Adrian W. Laxton, Stephen B. Tatter, Lance D. Miller, Roy E. Strowd, Aleksander Skardal

**Affiliations:** 1 Department of Biomedical Engineering, The Ohio State University, 140 W. 19th Avenue, Columbus, OH 43210, USA; sugavanamsivakumar.1@buckeyemail.osu.edu; 2 Wake Forest Institute for Regenerative Medicine, Wake Forest School of Medicine, 391 Technology Way, Winston-Salem, NC 27101, USA; stforsythe@gmail.com; 3 Department of Cancer Biology, Wake Forest School of Medicine, Medical Center Boulevard, Winston-Salem, NC 27157, USA; ldmiller@wakehealth.edu; 4 Department of Neurosurgery, Wake Forest School of Medicine, Medical Center Boulevard, Winston-Salem, NC 27157, USA; alaxton@wakehealth.edu (A.W.L.); statter@wakehealth.edu (S.B.T.); 5 Wake Forest Baptist Comprehensive Cancer Center, Wake Forest Baptist Medical Center, Winston-Salem, NC 27157, USA; 6 Department of Neurology, Wake Forest School of Medicine, Medical Center Boulevard, Winston-Salem, NC 27157, USA; 7 The Ohio State University and Arthur G. James Comprehensive Cancer Center, 460 W. 10th Avenue, Columbus, OH 43210, USA

**Keywords:** glioma, tumor constructs, 3D culture, hydrogel biomaterials, temozolomide

## Abstract

Glioblastoma (GBM) is an extremely aggressive and incurable primary tumor of the brain. GBM is characterized by interpatient and intratumoral heterogeneity, making this cancer particularly resistant to therapy and likely to recur. Mapping the complex dynamics that underpin the development and evolution of gliomas with human-based in vitro models is difficult. This study aimed to generate 3D glioma patient-derived tumor constructs (PTCs) using a clinically relevant, Matrigel-free, hyaluronic acid system, evaluate their suitability in drug screening assays, and determine the stability of their genetic profiles compared to originating tumors. In this study, we utilized a synthetically modified hyaluronic acid and gelatin hydrogel system to generate tumor constructs containing cells from clinical glioma biospecimens. PTCs were characterized phenotypically, after which they were deployed in chemotherapy drug screens using temozolomide (TMZ) and a P53 activator compound. Drug responses of these 3D cultures were compared with 2D cultures, as well as PTCs that were generated after passaging in 2D. RNA sequencing was used to evaluate genetic parity between PTCs or 2D cultures with originating tumor tissues, using The Cancer Genome Atlas (TCGA) GBM subpopulations for subcategorizing. PTCs were created successfully from five World Health Organization (WHO) grade 4, two grade 3, and two grade 2 gliomas. PTCs were maintained with high viability. Chemotherapy drug screens demonstrated that expected TMZ responses were observed for Isocitrate dehydrogenase (*IDH*) mutant diffuse gliomas while drug response was variable for *IDH* wildtype GBM PTCs. PTCs demonstrated stable drug response over time, while 2D passaging resulted in significant shifts in drug sensitivity. RNA sequencing revealed maintenance of subpopulation signatures for PTCs which clustered with their originating patient tumor tissue. In contrast, 2D cultures largely clustered together regardless of the patient. Our PTC approach utilizes a defined hydrogel biomaterial system that maintains the genotypic and drug response characteristics of patient tumors making this an ideal ex vivo model for translational applications.

## 1. Introduction

In the last decade, clinical trials have not resulted in successful therapeutics that meaningfully prolong the survival of patients with glioblastoma (GBM). Despite decades of brain tumor research, the average survival time following diagnosis of GBM has only increased from around 12 months to 20 months [1,2]. One of the major reasons for this failure to generate improved patient outcomes can be attributed to the lack of reliable in vitro GBM drug testing models which consistently reflect the complex tumor environment and heterogeneous genomic profile observed in vivo [3].

The emerging field of 3D patient-derived tumor models, such as organoids and other 3D tumor constructs, offers a tool to bridge this gap in GBM cancer modeling by serving as a reliable technique to study tumor heterogeneity and tumor-environmental contributors to gliomagenesis, tumor response, and treatment resistance. These various tumor models are generally small-sized 3D tissue constructs—generally ranging from 50 μm to 4 mm or so—which mimic the corresponding in vivo tissue and can be employed in a variety of pre-clinical applications including cell proliferation, drug assays, and the intratumoral evolution of cellular composition and genomic profiles that occur in response to drug insults or spontaneously [4,5,6,7].

The landmark study on the generation of physiologically accurate 3D in vitro models were in the form of gastrointestinal organoids from adult stem cells by Sato et al., which was published in 2009, after which there has been explosive growth in the field of organoid-based research and other alternative 3D in vitro models [5,8]. Such 3D models have become powerful tools in areas of research involving disease modeling, precision medicine, toxicology, and regenerative medicine [9,10]. In the last few years, improved biomaterials and microfluidic organ-on-a-chip technologies have enabled the generation of 3D tumor models from a wide variety of tumors isolated from patients. CRISPR/CAS9 technologies have facilitated engineering genetic alterations into non-malignant organoids to create tumor organoids with specific genetic profiles [11]. Patient-derived tumor organoids (PTOs) and PTCs have since been employed in predicting response to chemotherapy in correlation to genetic differences unique to individual patients [12]. Numerous studies have been published to show that these models can predict therapy response to chemotherapy in patients [13,14]. In recent years, our lab has employed a bioengineered hyaluronic acid (HA) and collagen/gelatin-based hydrogel bioink platform as a defined organoid culturing system alternative to Matrigel [15,16,17]. While Matrigel is the most widely used biomaterial for tumor organoids it is not well-defined. It contains countless growth factors, cytokines, and ECM components that are not defined and can serve as confounding variables. Alternatively, our HA-based system is completely defined, enabling increased experimental control over tumor models generated with this biomaterial. With this platform, we have demonstrated its utility in creating PTCs for chemotherapy and immunotherapy screening applications in colorectal, lung, melanoma, mesothelioma, appendiceal, sarcoma, adrenocortical carcinoma, and recently, glial tumors [15,16,18,19,20,21,22,23,24,25,26].

In vitro patient-derived GBM models have made their successful foray into glioma research in the last few years in the form of PTOs, beginning with work by the Rich lab [27]. Studies have shown that GBM PTOs are a faithful in vitro model of GBM. They preserve the cellular and mutational diversity of the parent tumor. GBM PTOs can be used to study intra and intertumoral heterogeneity that faithfully recapitulates the histological features, cellular diversity, and genomic profiles of parental tumors used to generate the PTOs. This has been recently verified by immunohistology, single-cell transcriptomics, and mutational analysis characterization [28]. GBM PTOs have also been used to recreate intrinsic immune landscapes unique to patients, and these tumor microenvironments have been deployed in testing immune-oncological targets [29] in a similar manner to the approach our laboratory employed in several other cancers [21,22,30]. These organoids can be consistently generated to employ precision medicine approaches that investigate patient-specific therapeutics [16,18,19,20,21,22,31]. GBM organoids have also been biobanked and then used subsequently to generate xenografts and long-term cultures for targeted drug testing and CAR-T co-culture [32]. This represents significant progress in glioma organoid utility, but also fails to address a key limitation, which is rapid generation of clinically relevant data that could have clinical implications. The status quo in PTO research is that the organoids need to be maintained and passaged in vitro for months in basement membrane extract (BME) biomaterials, such as Matrigel, before considered true organoids. Patients do not have that amount of time in many cases. Rapid turnarounds from PTO establishment to actionable empirical drug screening data and genetic sequencing data could add a useful tool to clinical oncology. Moreover, BMEs are derived from murine sarcoma tumors, which could push GBM cell phenotypes and genotypes away from their initial states due to signaling from ECM and paracrine signals from murine sarcoma ECM.

One application of patient-derived tumor models is the study of intratumoral heterogeneity, the presence of genetically or regionally distinct clonal and subclonal differentiated tumor and cancer stem cells in the same patient [33]. This is different from interpatient heterogeneity, in which tumors differ between different cancer patients. Branched clonal evolution in GBM contributes to the selection of resistant clones from residual tumor cells after resection and adjuvant therapy [34]. Recurrent GBM frequently exhibits a genetic profile that differs from the primary tumor and may demonstrate a varying response to treatment. Even treatment regimens that were highly efficacious in a primary GBM tumor are often ineffective at recurrence due to the shifts in the tumor’s genomics [35]. This intratumoral heterogeneity and the constantly evolving genetic profile are major impediments to the effective clinical management of these patients [36].

Herein, we address a translational gap in how GBM PTCs—not PTOs—generated with well-defined biomaterials can be used to understand intra and interpatient heterogeneity by describing deployment of GBM PTCs for drug response studies and robust characterization. Other GBM organoid models do exist, but most are supported by undefined BME-based biomaterials, while our platform is defined, minimizing biomaterial-based potential confounding variables [37]. These experiments build upon the existing literature of GBM PTOs and use this approach to model clonal evolution and treatment resistance. Using our well-characterized hyaluronic acid-based hydrogel system we created GBM PTCs from GBM patient-tumor biospecimens. These PTCs were characterized by evaluating the viability of PTC cultures over time, compared to 2D cultures and studying the evolution of tumor subpopulations over time and in response to treatment. Following the evaluation of phenotypic heterogeneity, we demonstrate the utility of these PTCs in chemotherapy screening assays, with a focus on how traditional 2D cell culture drives a shift in drug responses. Lastly, genomic sequencing data was used to evaluate the capability of our hydrogel platform to maintain PTCs with genetic profiles similar to those of the originating tissues. Ultimately, herein we demonstrated that a defined, HA-based hydrogel biomaterial system is capable of generating PTCs that represent the tumor tissue from where they were derived, and this fact can have future implications in clinical applications.

## 2. Materials and Methods

### 2.1. Glioma Biospecimen Acquisition and Processing

The tumor tissue biospecimens were obtained from patients undergoing tumor resection surgeries at the Wake Forest Baptist Comprehensive Cancer Center in adherence to the approved Institutional Review Board protocol (WFUHS IRB #00049693). Glioma classification was based on the WHO 2016 CNS Classification of Central Nervous System Tumors. In the operating room, freshly removed biospecimens were immediately placed in sterile RPMI (Hyclone, Logan, UT, USA) media in sterile containers. Samples were placed on ice and immediately transferred to the laboratory in most cases. However, due to timing, several biospecimens in RPMI were maintained at 4 °C overnight, after which biospecimens were transferred to the laboratory. The biospecimens were washed in phosphate-buffered saline (PBS) (Lonza, Morristown, NJ, USA) with 5 µg/mL gentamicin (Sigma, St. Louis, MO, USA) + 1 µg/mL amphotericin B (Sigma) + 10 µL/mL penicillin and streptomycin (Thermo Fischer, Waltham, MA, USA). The tumor specimen was removed from any tissue debris and placed in Dulbecco’s Modified Eagle’s Medium (DMEM) (Hyclone) with 2% penicillin-streptomycin (Thermo Fischer) for further processing. Tumors were minced and placed into low glucose DMEM with 160 µL/mL collagenase (Vitacyte, Indianapolis, IN, USA) HA + 40 µL/mL BP protease (Vitacyte) for up to two hours on a shaker plate at 37 °C. The tissue digestion solution is neutralized with an equal amount of cold supplemented serum supplemented DMEM high glucose. The digested tumors were then filtered through a 100 µm cell filter and centrifuged to create a cell pellet. The pellet was resuspended in 1 mL of BD Pharm Lyse with 9 mL of deionized water for 5 min (BD Biosciences, San Diego, CA, USA) to remove the red blood cells from the sample. The conical was filled to 50 mL with deionized water and centrifuged, after which the lyse buffer with lysed cells was aspirated, and the remaining pellet was resuspended. The dead cell removal kit (Miltenyi Biotec, Auburn, CA, USA) was used to remove the dead cells from the tumor cell suspension. Lastly, the final cell number was determined ahead of use.

### 2.2. Extracellular Matrix Hydrogel Preparation and PTC Biofabrication

HyStem-HP hydrogels (ESI-BIO, Alameda, CA, USA) were employed to suspend the cells isolated from the tumors in order to create the PTCs. This hydrogel system was chosen because the primary component is hyaluronic acid which is the major extracellular protein in the brain, and we have deployed it in multiple PTC applications previously [18,19,21,30,38]. Moreover, this is a defined system, unlike basement membrane extract hydrogels derived from murine tumors, in which countless potential confounding variables exist [39]. The components of the hydrogel include thiol-modified HA with heparin pendant chains (Heprasil), thiol-modified gelatin (Gelin-S), and a thiol-reactive PEGDA cross-linker (Extralink; molecular weight of 3400 kDa). Heprasil was reconstituted with distilled water to produce a 1% weight by volume solution and both Gelin-S and Extralink were reconstituted with 0.5% photoinitiator dissolved in water (Irgacure D-2959, Sigma Aldrich, St. Louis, MO, USA) to produce 1% weight-by-volume (*w*/*v*) solution. The reconstituted vials were placed in a 37 °C incubator for 40 min for the solids to completely dissolve and form clear solutions. The hydrogel precursor solution was prepared by mixing Heparasil, Gelin S, and Extralink in the ratio of 1:1:0.5 by volume. Ten µL of this premixed solution was used to fabricate PTCs of 20 million cells/mL in 48-well plates previously coated with a thin layer of polydimethylsiloxane (PDMS) to form consistently round droplets, which were subsequently photocrosslinked by two seconds of ultraviolet light exposure (365 nm, 18 W/cm [2]). Each PTC then received 500 µL DMEM media (Hyclone), which was refreshed every 3 days during PTC culture.

Hydrogel droplets used to form PTCs were generally deposited using a 10 µL pipette. However, as proof of concept some PTCs were 3D bioprinted using a BIO X bioprinter (CellInk, Gothenburg, Sweden). In this case we utilized the same hydrogel described above that we have previously used in bioprinting applications [40]. In those cases, a syringe containing the hydrogel precursor with cells was fitted with a 22 G × 1/2′′ blunt tip needle and loaded into the bioprinter equipped with a syringe pump printhead. When ready to print, the 48-well plate was placed in the BIO X printer’s print stage, and the printer was then homed and calibrated so that the organoids were made in the center of each well. The onboard droplet printing method (built in software) was used to create the PTCs approximately 10 µL in volume, at an extrusion rate of 5 µL s^−1^. Once printed, the plate was exposed to ultraviolet (UV) light, as above, to crosslink the PTCs.

### 2.3. Cell Culture and Propagation

For 2D cell cultures, cell suspensions from the processed biospecimens containing 1 million cells were seeded onto a poly-L-lysine (PLL)-coated 150 mm surface tissue culture treated immediately following tumor cell dissociation. The cells (BT1 to BT8) were maintained in astrocyte media with astrocyte growth supplement and 10% fetal bovine serum (Sciencell, Carlsbad, CA, USA) with media replenishment every 3 days. The cells from BT9 were maintained in DMEM high glucose (Hyclone) supplemented with 10% FBS (Hyclone) and 2% streptomycin and penicillin (Thermo Fisher) with media replenishment every 3 days. GBM cell line, A172 (ATCC^®^ CRL-1620TM) was obtained from ATCC, (Manassas, VA, USA) and was cultured in Dulbecco’s Modified Eagle Medium (DMEM)—high glucose (Hyclone) with 10% fetal bovine serum (FBS) (Thermo Fischer), 1% L-glutamine (Thermo Fischer), and 1% penicillin/streptomycin (Thermo Fischer) in a tissue culture incubator at 37 °C with 5% CO_2_. Cells were collected from tissue culture plates for subsequent studies using Trypsin/EDTA (Thermo Fisher). The cultures were allowed to attain 90% confluency after which they were passaged. Cells were dissociated from tissue culture dishes using 0.5% Trypsin-EDTA for subsequent assays.

### 2.4. PTC Viability

Relative metabolic activity was quantified as an indicator of cellular response to drug treatments by adenosine triphosphate (ATP) activity quantification assays. ATP assays (CellTiter Glo 3D; Promega, Madison, WI, USA) determined relative cell number by quantification of cellular ATP. Luminescence values were determined on a Varioskan LUX multimode microplate reader M5 (Thermo Fisher) tunable plate reader system. LIVE/DEAD staining (LIVE/DEAD Viability/Cytotoxicity Kit for mammalian cells, Thermo Fisher) was performed on day 10 at the end of the drug exposure period (3 days in total). Spent media was first aspirated from wells, after which a 100 mL mixture of PBS and DMEM (1:1) containing 2 mM of calcein-AM and 2 mM of ethidium homodimer-1 was added. Constructs were incubated for 60 min after which spent media was again aspirated and replaced with clean PBS. Fluorescent imaging was performed using a Leica TCS LSI macro confocal microscope (Leica Microsystems, Wetzlar, Germany) to generate maximum projections from the captured z-stacks representing the 3D constructs.

### 2.5. Histology and Immunohistochemistry

After 10 days in culture, PTCs were removed and fixed in a 4% paraformaldehyde solution (Electron Microscopy Services, Hatfield, PA, USA) at 4 degrees for 24 h. Fixed PTCs and tissue were then histologically processed, embedded in paraffin, and sectioned to produce slides for imaging. Hematoxylin and eosin staining was performed to determine cell size and position in the PTCs and tissue.

Immunohistochemistry (IHC) was performed to analyze the biomarker presence of IDH1 R132H, GFAP, KI67, EGFR, OLIG2, AND P53 on sectioned slides. Antigen retrieval was performed with either proteinase K (ab64220, Abcam, Cambridge, UK) or heat-mediated antigen retrieval with a pH 6 citrate buffer (ab93678, Abcam) on all slides prior to staining. Primary antibodies for IDH1 R132H (DIA-H09-M,1:50, Dianova, Hamburg, Germany), Ki67 (ab238020, Abcam, 2 μg/mL dilution), GFAP (Invitrogen, Carlsbad CA USA, PA5-16291, 1:50), P53 (ab1101, Abcam, 5 μg/mL), EGFR (ab52894, Abcam, 1:100) and Olig2 (ab109186, Abcam, 1:100) were incubated on slides overnight at 4 °C. The mouse and rabbit specific HRP/DAB (ABC) Detection IHC Kit (ab64264, Abcam) was used to create chromogenic images and the protocol was followed according to manufacturer’s instructions.

### 2.6. Drug Screening Studies

The PTCs (or 2D cultures) were then employed in drug screening assays with TMZ and an experimental p53 pathway activator drug NSC59984 (S1237 and S8106, respectively; Selleckchem, Houston, TX, USA). Stock solutions of 100mM were prepared from the drug by dissolving the drug compounds in DMSO. Drug stock solutions were diluted with DMEM cell culture media to the following concentrations: TMZ—100 µM, 500 µM, and 1 mM; and NSC59984—10 µM and 100 µM. For drug screening assays, PTCs were maintained in drug-free culture conditions for 7 days, after which media was aspirated from the wells and fresh media containing the drug concentrations described above were administered to the PTCs. PTCs were maintained for 3 days before assessing the effect of the drugs on these constructs using ATP quantification and LIVE/DEAD staining as described above.

For several samples in which cell yield was high, cells freshly isolated from the biospecimens (passage 0, or P0) were passaged once (P1) or twice (P2). These P1 and P2 cell populations were then used to form P1 or P2 PTCs or plated at 10,000 cells per well in 48-well plates for 3 days in drug-free media and at 70% confluence subjected to the drug screen for 2 days as described above to evaluate the effects of passaging cells in 2D tissue culture conditions on drug response. ATP quantification and LIVE/DEAD staining were employed as described above.

### 2.7. P21 Expression Assay

A172 constructs were fabricated with the same method as the PTCs as mentioned above. Drug treatment with P53 activator NSC59984—100 µM on day 7 of culture of A172 constructs was performed like PTC constructs and on day 10, the constructs were processed for RNA extraction. Total RNA of A172 constructs was extracted using Trizol (Thermo Fischer Scientific, Waltham, MA, USA) as per manufacturer’s protocol followed by cDNA synthesis with Superscript IV VILO Master mix (Thermo Fischer Scientific) with ezDNase to remove genomic DNA. Following reverse transcription, quantitative PCR was performed using QuantStudio™ 3 Real-Time PCR system (Thermo Fisher Scientific), and taqman primers used in this study are listed in the table. Fold changes were calculated by subtracting the Cq values of the control human GAPDH from the Cq values of the gene of interest. The relative gene expression was reported as ΔΔCt values, where ΔΔCt = ΔCt_treatment_ − average(ΔCt_control_).

### 2.8. Expression Profiling Analysis

RNA sequencing (RNAseq) was performed on 24 flash-frozen specimens derived from 6 patients in the Cancer Genomics Shared Resource (CGSR) of the Wake Forest Baptist Comprehensive Cancer Center. Patient specimens included dissociated primary tumor cells (P0; “Tu”), PTC control (P1; “PTC”) and drug-treated specimens (“PTC_T”), and corresponding 2D cultures (“Pla”). Frozen specimens were homogenized on a Bead Ruptor Bead Mill (Omni International, Kennesaw, GA, USA) and extracted for total RNA using the Qiagen AllPrep DNA/RNA kit. RNA quality was subsequently assessed by electrophoretic tracing (Agilent Bioanalyzer, Santa Clara, CA, USA), and cDNA libraries were constructed using the Takara SMARTer^®^ Stranded Total RNA-Seq Kitv2 (Takara Bio, Kusatsu, Japan), according to the manufacturer’s instructions. Size distributions of libraries were quality checked on an Agilent 2100 Bioanalyzer, and library quantity was measured on a Qubit 3.0 (ThermoFisher, USA). Dual-indexed libraries were pooled and sequenced to a target read depth of 25 M reads per library using 100 bp single-end sequencing on an Illumina NovaSeq 6000 SP-100 flow cell. For all samples, >80% of sequences showed >Q30 Phred quality scores (FASTQC analysis, Babraham Bioinformatics, Brabraham Institute, Cambridge, UK). Excess adapter sequences were removed with Trimmomatic [41] version 0.32. Reads were aligned to the GRCh38 reference human genome (https://www.ncbi.nlm.nih.gov/assembly/GCF_000001405.39, accessed on 29 June 2020) using the STAR sequence aligner [42], and gene counts were determined using the featureCounts program [43] version 2.0.3. Normalized gene expression estimates were computed using the DESeq2 Bioconductor package [44] and assigned to the glioblastoma subtypes defined by Verhaak and colleagues [45] as follows. The training matrix from that report, comprising 173 GBM expression profiles (TCGA_unified_CORE_ClaNC840.txt; downloaded from https://gdc.cancer.gov/about-data/publications/gbm_exp), was used to train the ClaNC nearest centroid-based class predictor [46] as described [45]. Our normalized gene expression matrix was then integrated with the brain tumor training matrix via ComBat batch correction [47], and the trained centroid classifier was applied to our samples for subtype assignment.

## 3. Results

### 3.1. Experimental Design and Workflow

**Figure 1:**
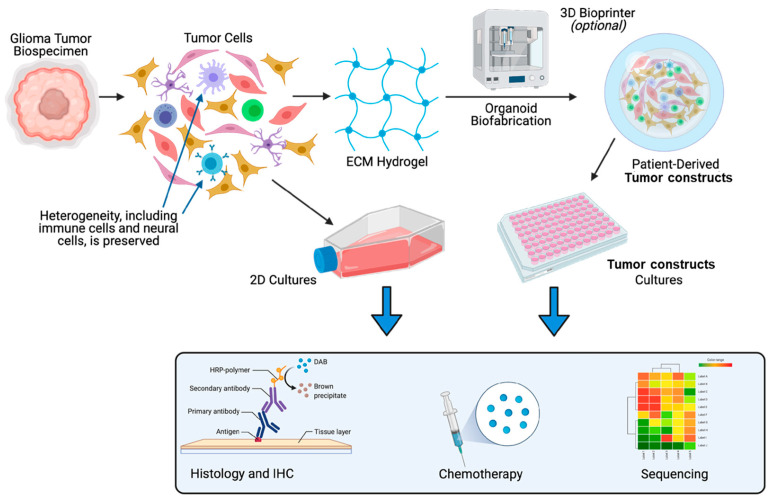
Overall GBM PTC study design. Biospecimens were dissociated and cells were combined with our ECM hydrogel to generate 3D GBM PTCs. Alternatively, cells were plated on 2D tissue culture plastic. Cultures were then subjected to characterization studies, chemotherapy studies, and RNA sequencing.

Our overall goal has been to demonstrate that one can generate glioma PTCs that are viable and amenable to subsequent assays such as drug screens that can be employed in a diagnostic manner to aid in optimizing therapeutic decisions for specific patients. Our PTC platform is supported by a customizable hyaluronic acid-based hydrogel, which can be combined with synthetically modified gelatin, collagen, fibronectin, laminin, and other ECM components [48]. Therefore, as we outline in Figure 1, we undertook a rigorous evaluation of this approach for creating and deploying GBM PTCs as alternatives to BME-based PTOs, including biomarker evaluation, drug screening assays, RNA sequencing, and comparison between our approach and more traditional 2D culture methods.

### 3.2. Glioblastoma Biospecimen Procurement to PTC Biofabrication

Twenty-nine patient-tissue biospecimens were procured. Successful PTC fabrication was performed for sixteen specimens, while the others were banked for future studies. Several of these were determined to be brain metastases or medulloblastomas, and as such were removed from this study. Due to the low yield of several tumor tissues, for example, due to necrotic regions, eight of the specimens did not yield enough cells for fabrication of sufficient numbers of PTCs for all of the subsequent experiments. In general, biospecimens were transferred to the laboratory for processing within 1 or 2 h following resection. The processed specimens with which all experiments in this study were performed comprised nine gliomas, including five glioblastomas (IDH wild type, WHO Grade 4), three astrocytomas (IDH mutant, WHO Grade 2–3), and one oligodendroglioma (IDH mutant, 1p19q codeleted), according to the 2021 Classification of CNS Tumors [49]. Nomenclature for each biospecimen PTC set, along with diagnosis, clinical evaluation of IDH status, MGMT methylation, P53 status, and other clinical descriptors are described in Table 1. In addition, biospecimen mass, cell yield, and general drug responses are included in Table 1.

### 3.3. Glioma PTC Viability and Characterization

**Figure 2:**
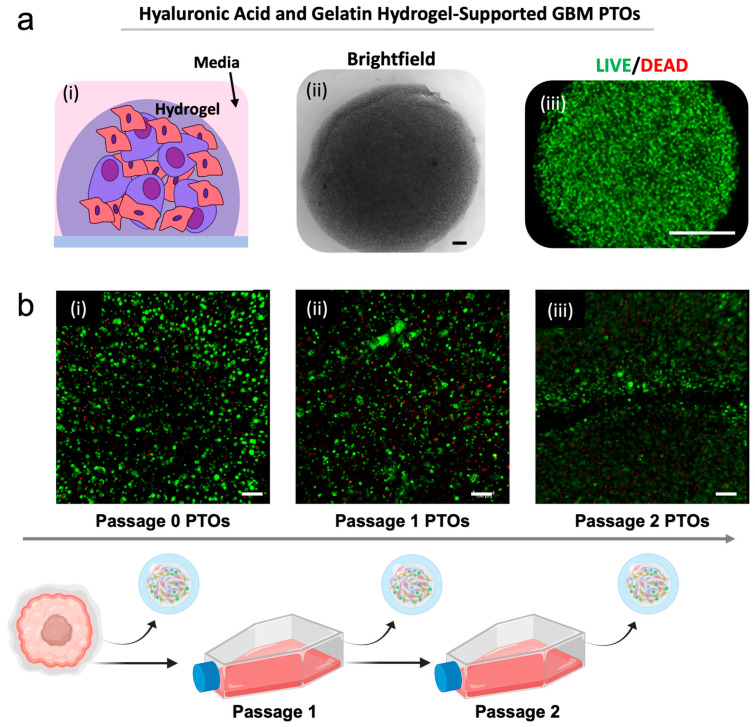
PTC biofabrication results in highly viable cultures that can be passaged and maintain key glioma cell subpopulations. (**a**) Glioma PTCs exist as heterogeneous cell subpopulations suspended together in an ECM hydrogel comprising thiolated hyaluronic acid and gelatin, crosslinked by PEGDA (**i**). (**ii**) Brightfield (scale bar—100 μm) and (**iii**) fluorescent (LIVE/DEAD) (scale bar 250 μm) microscopy images of glioma PTCs. (**b**) Passaging of cells on tissue culture plastic allows for highly viable PTCs at several passages: (**i**) passage 0, (**ii**) passage 1, and (**iii**) passage 2, as seen by LIVE/DEAD staining. Scale bar: 100 µm. Green—calcein AM-stained viable cells; Red—ethidium homodimer-1-stained dead cell nuclei.

Rather than cell aggregates that spontaneously form in Matrigel, our GBM PTCs exist as cells suspended in a covalently crosslinked hyaluronic acid and gelatin hydrogel (Figure 2a(i)). These PTCs, seen under brightfield in Figure 2a(ii) are highly viable following PTC fabrication (Figure 2a(iii)). After establishment, PTCs underwent a set of characterization assays. First, to confirm viability in PTCs and that these cells could be propagated successfully in vitro, viability assays were performed at several passages. LIVE/DEAD staining demonstrated robust cell viability in the PTCs generated from cells at passage 0, passage 1, and passage 2, with high numbers of viable cells (green) and few dead cells (red) (Figure 2b). We should be clear that in this experiment, the cells from the originating biospecimen were propagated in 2D, from which PTCs were generated. In other words, the cells were not passaged in 3D using Matrigel which can be diluted to retrieve cell clusters. While we have demonstrated the capability to passage tumor cells in our HA-based hydrogels, it is more time-intensive, requiring the dissociation of the hydrogel itself.

Immunohistochemistry (IHC) staining of PTC sections generated from grade 4, 3, and 2 glioma biospecimens showed positive expression of a panel of biomarkers associated with gliomas. IDH1 R132H and EGFR were expressed, which are common in diffuse gliomas and GBM, respectively (Supplementary Figure S1a–c(i,ii)). Ki67, a biomarker for proliferation, was expressed throughout samples (Supplementary Figure S1a–c(iii)), indicating that cells were proliferating within PTC cultures. GFAP (glial fibrillary acid protein), a biomarker associated with astrocytomas, was also observed (Supplementary Figure S1a–c(iv)). P53 was also positively expressed in general (Supplementary Figure S1a–c(v)). Lastly, OLIG2 is a transcription factor expressed widely in diffuse gliomas, which was observed in our PTCs (Supplementary Figure S1a–c(vi)). These markers indicate PTC preservation of expected cell types in gliomas.

### 3.4. Passaging Causes Drug Response Shifts

**Figure 3:**
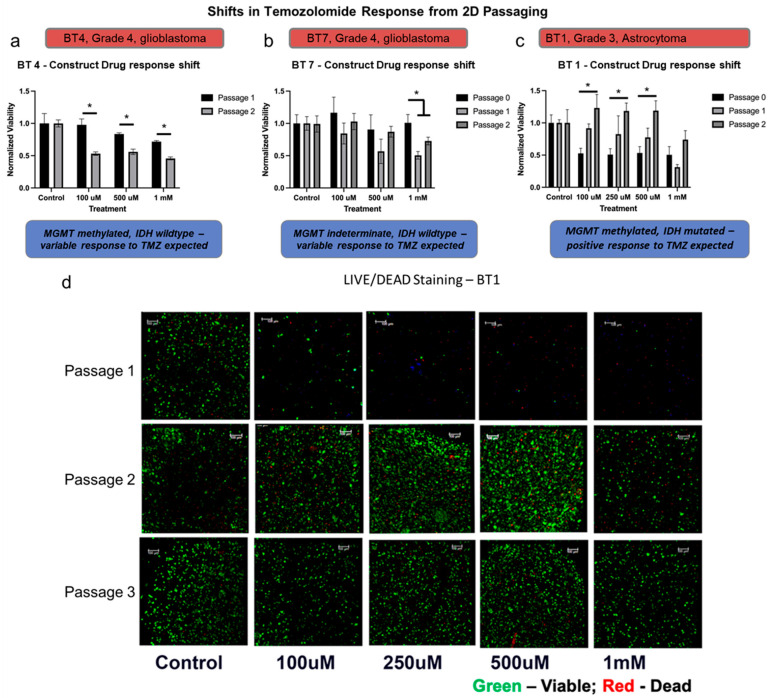
Passaging of glioma cells in 2D culture drives shifts in drug sensitivity in PTCs. (**a**–**c**) ATP activity quantification showing temozolomide response differences between passages of glioma PTCs BT4, BT7, and BT1. (**d**) Maximum projection fluorescent microscopy images of LIVE/DEAD-stained BT1 PTCs. Statistical significance: * *p* < 0.05. Green fluorescence—calcein AM-stained viable cells; Red fluorescence—ethidium homodimer-1-stained dead cell nuclei. Scale bars—100 µm.

Patient-derived cancer cells cultured in traditional 2D cell culture were once considered an adequate model to test pharmacological responses that can recapitulate drug responses in human patients, but studies in recent years have demonstrated that these cells undergo profound phenotypical changes and differed drug responses than those observed in the patients [50]. To compare drug response with 3D PTC and 2D cell culture, drug response via cell proliferation assay was compared between PTCs and 2D cultures fabricated from the same patient cells but after passage 1 and passage 2 on a 2D plastic cell culture substrate to yield P1 PTCs and P2 constructs respectively. The drug response of BT7 and BT4 PTCs fabricated after cell isolation has no response to drugs but P1 PTC (i.e., one cell culture passage) has acquired a response to 1mM temozolomide and this sensitivity is maintained in P2 PTC of BT4 and BT7. Increased proliferation is seen with 100 µM temozolomide in both PTCs. The BT1 PTCs had a response to all the concentrations of temozolomide tested but P1 PTCs had a response only to temozolomide at 1mM whereas P2 PTCs had lost sensitivity to all the concentrations of temozolomide (Figure 3). The drug study was repeated in a 2D cell culture of BT7, and the drug response of passage 1 cells was compared to the drug response of passage 2 cells. It can be observed from Supplemental Figure S2 that the trend of cells gaining resistance to temozolomide as the passage number increases is much more pronounced in this 2D drug study. As shown in Figure 3d, we were able to passage BT1 further to passage 3 in which similar drug resistance was observed like in passage 2 PTCs. However, our goal was not to create future populations of cells. Our goal was to utilize these cells close to time of procurement.

### 3.5. Patient-Specific GBM Tumor Construct Chemotherapy Treatment and Response

**Figure 4:**
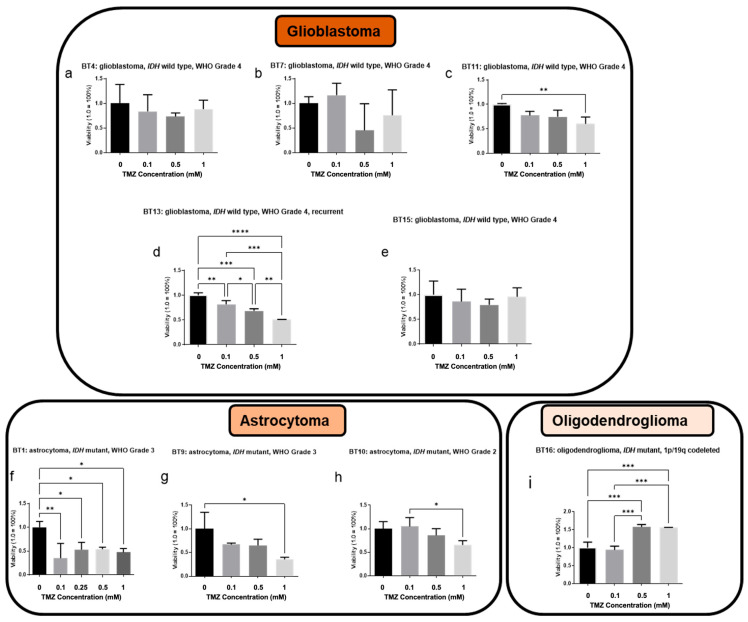
Temozolomide (TMZ) chemotherapy screens, stratified by grade, show that GBM PTCs have a variable response, while grade III and grade II gliomas are largely more responsive to TMZ therapy. ATP activity quantification is proportional to PTC viability. Statistical significance: * *p* < 0.05; ** *p* < 0.01; *** *p* < 0.005; **** *p* < 0.001.

**Figure 5:**
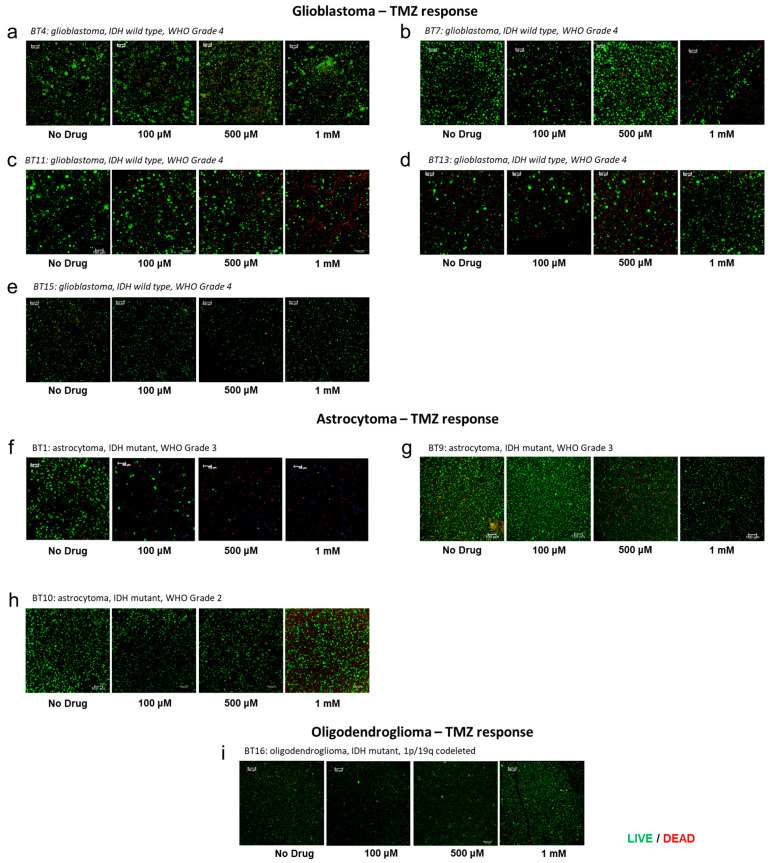
Treatment of glioma PTCs with temozolomide has efficacy in most samples as evidenced by decreases in viable cells and increases in dead cells. This becomes less evident in astrocytomas and the oligodendrogloma PTCs. Green fluorescence—calcein AM-stained viable cells; Red fluorescence—ethidium homodimer-1-stained dead cell nuclei.

The current chemotherapy-based standard of care for GBM following surgical resection is treatment with TMZ [51]. Drug responses following TMZ treatment have been stratified based on the grade of the tumor. In Figure 4, the drug response of GBM (IDH wt, grade 4) PTCs to the different concentrations of TMZ can be observed. The 1 mM showed modest response in BT11 and BT13 PTCs, which were sensitive to all three doses whereas BT4, BT7, and BT15 PTCs exhibited no sensitivity to temozolomide therapy. Despite the predictive value placed on MGMT promoter methylation status in high-grade glioma, it should be noted that the MGMT promoter methylation is only one aspect of a complex biological system and recent studies have demonstrated that the MGMT promoter methylation status does not correlate with patient temozolomide response [52,53]. LIVE/DEAD image results support this conclusion (Figure 5). The drug response of these GBM PTCs to a drug that activates p53 activation in cells through induction of mutant p53 protein degradation and p73 activation is shown in Supplemental Figure S1. To assess potential off-target effects of NSC59984, we tested the compound in A172 glioblastoma constructs, the cell line that retains wild-type p53 [54]. Following treatment, A172 cells exhibited increased p21 expression, consistent with activation of a canonical p53-dependent transcriptional response as seen in Supplemental Figure S5. These findings indicate that NSC59984 does not exert nonspecific cytotoxic effects in this context but instead engages the p53 pathway, thereby supporting the specificity of its mechanism of action observed in mutant p53-harboring cells [55]. Again, it can be observed that there is no correlation between MGMT status and drug sensitivity. Similar to temozolomide, BT15 PTCs have no response to this drug. All other glioma PTCs including BT4, BT7, BT11, and BT13 have a dose-dependent response.

In astrocytoma tumors, all PTC sets were sensitive to TMZ at 1 mM. However, only one grade 3 (BT1) tumor was sensitive to the other doses tested while the other two tumors showed no effect (BT9 and BT10). In fact, there was a small increase in viability at 100 µM for BT10 PTCs, which was abrogated at high dose treatment. In the oligodendrocytoma-derived PTC set B16, there was no response to temozolomide treatment. The p53 activator has a dose-dependent response in grade 2 astrocytomas and in grade 3 astrocytomas. BT10 PTCs also had a dose-dependent response to p53 activator, whereas the BT16 PTCs exhibited no response to the drug (Supplemental Figure S3).

### 3.6. 3D Constructs Outperform 2D Tissue Culture at Preserving Gene Expression-Based Subtype Designation of the Originating Tumor

**Figure 6:**
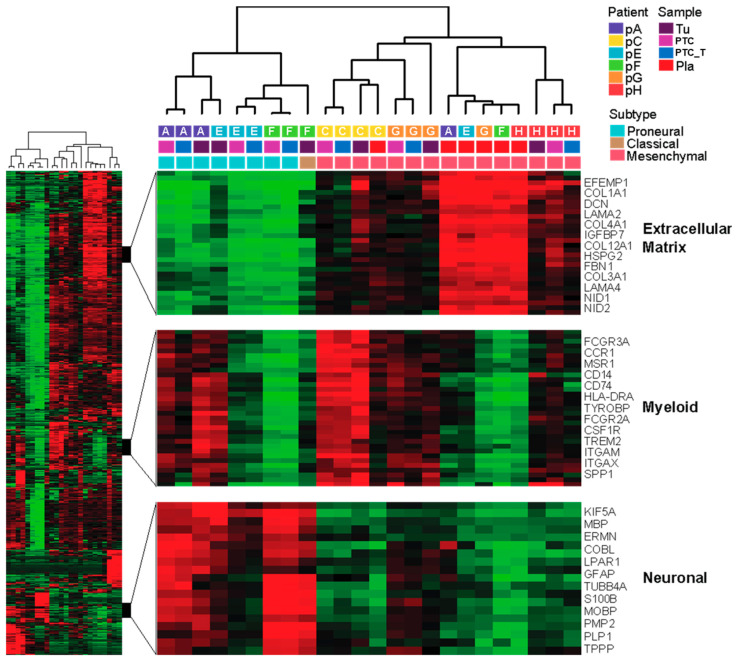
RNAseq shows that ECM hydrogel PTCs preserve genomic profiles of glioma biospecimen-derived cells. RNAseq hierarchical cluster analysis of originating tumor cells (Tu), PTCs (PTC), temozolomide-treated PTCs (PTC_T), and cells maintained in 2D tissue culture (Pla), from six human patients (A, C, E, F, G, and H). Tu, PTC, and PTC_T cluster together by the patient, while Pla cultures cluster together, regardless of the patient. PTC/PTC_T culture preserves the initial majority GBM subtype, while Pla promotes shifts to a mesenchymal subtype. Heatmap colors: Green represents lower differential expression, while red represents higher differential expression. Black represents no differential expression.

We performed genome-wide RNAseq analysis on the primary tumor, 3D PTC, TMZ-treated 3D PTC, and 2D culture specimens. To investigate inter-sample transcriptomic variation, we selected the top 1000 genes that showed the greatest magnitude of inter-sample variation in expression (i.e., the genes with the highest standard deviation values) and performed unsupervised hierarchical clustering on the specimens using average linkage clustering (with Pearson correlation as the distance metric). As observed from the specimen dendrogram (columns) in Figure 6, most specimens formed subclusters that correlated strongly with a patient of origin. However, this result was not consistent across specimen types. While patient-matched parental tumor (Tu) and 3D organoid specimens (Org and Org_Tx) tended to cluster near one another, specimens derived from 2D cultures on plastic (Pla) tended to cluster together, showing greater transcriptomic similarities with one another than with their corresponding patient-matched counterparts. In parallel, we used the method of Verhaak and colleagues [45] to assign each specimen to its corresponding molecular subtype (Proneural, Neural, Classical, or Mesenchymal). We observed that the primary hierarchical branches of the dendrogram robustly dichotomized the Proneural and Mesenchymal specimens. Moreover, while subtype designation was largely conserved between parental tumor and patient-matched organoid specimens, all Pla specimens were classified as Mesenchymal, including those that corresponded with patient-matched tumor and organoid specimens of Proneural or Classical subtype. Together, these findings are consistent with the ability of 3D constructs, but not conventional 2D cultures, to preserve tumor molecular subtype, while 2D cultures may skew non-mesenchymal tumor subtypes toward an artificial mesenchymal phenotype.

## 4. Discussion

Despite enormous efforts over the past several decades in cancer research to better define the heterogeneity of gliomas and identify actionable targets of intervention, little has been gained in terms of improved clinical efficacy. One hurdle impeding potential progress is a lack of suitable in vitro models, including low-grade gliomas which can be difficult to reliably culture and GBM which is the most lethal. GBM cell lines have poorly modeled drug response due to genetic, epigenetic, and microenvironmental drift. Capturing the heterogeneity observed in clinical GBM is critical.

Glioma science in the context of 3D in vitro models is not in its infancy and recent studies have demonstrated the feasibility of hyaluronic-based approaches to developing high-throughput platforms [56,57]. Recent studies demonstrated feasibility of glioma organoid biobanking [32], ability to characterize tumor-microenvironmental interactions that contribute to gliomagenesis and invasion [58], utility of testing personalized therapies by correlating glioma organoid mutational profiles with drug response including immunotherapy [32], and a recent paper from one group has suggested feasibility in establishing lower-grade glioma organoids, although the organoids described in this study are essentially heterogeneous cell spheroids cultured in ultra-low adhesion well plates, rather than ECM. Instead, cell culture media formulations drive success [59]. Despite this early work, translational gaps remain in GBM in vitro model science in neuro-oncology, including the low maturation of some glioma organoid systems, particularly for lower-grade and *IDH* mutant gliomas, a lack of a complete tumor microenvironment [4], and a lack of a reliable vascular network [60]. In this study, we address three important gaps in glioma in vitro model biology: (1) establishment of a glioma PTC platform based on defined biomaterial components, (2) the successful use of PTCs across the range of glioma histologies, WHO grades, and IDH mutational status, and (3) the evaluation of translational relevance of our 3D models versus 2D models, thus enabling more reliable interpretation of in vitro studies in glioma research and clinical practice.

First, we present an approach to generating glioma PTCs using an easy-to-deploy, well-defined hyaluronic acid and gelatin hydrogel system. Our approach avoids the utilization of the ubiquitously employed Matrigel organoid generation approach, which while effective due to its potency, is problematic due to its undefined composition which can introduce countless confounding variables into experiments. Moreover, as organoid and 3D cell cultures continue to advance towards applications such as clinical diagnostics or culturing of cells for cell therapy, materials such as Matrigel, being derived from murine sarcoma, is a non-starter in such clinical and translational applications while generation of PTOs using ULA plates is inconsistent and time-consuming. We show definitively that other approaches using defined materials can be effective alternatives. Here, we were able to generate glioma PTCs that were easily deployed in a variety of subsequent experiments, including patient-specific drug screening assays of astrocytic and oligodendroglial tumors, low and high-grade gliomas, and for both *IDH* mutant and wildtype specimens.

The drug screening assays we performed yielded data within clinical expectations. We observed a heterogeneous response to TMZ in GBM PTCs. Statistically significant positive responses were only demonstrated in 2/5 PTC sets. In comparison, 3/4 grade II or III glioma PTC sets responded to TMZ. We should note here that BT16 did not respond to TMZ. The BT16 specimen was obtained from a patient with an incidentally discovered grade 2, *IDH* mutant, MGMT promoter methylated oligodendrocytoma which did not require treatment and has remained without recurrence after surgery for >5 years. The lack of drug response may reflect the benign nature of this tumor at the time of tissue procurement.

However, perhaps more interesting than successful deployment into such assays is the observation that simply transferring patient-derived cells into 2D culture causes the cells to often undergo genetic drift causing changes in drug sensitivity as evidenced by our genomic analysis of PTCs, 2D cultures and originating tumor tissue, and altered drug responses of different passages of the corresponding patient-derived cells. Traditional cancer cell culture in 2D has been a widely used and crucial basic research tool that has been instrumental in gaining important knowledge in cancer biology, disease mechanisms involved in tumor progression, and drug discovery and development. However, it is now apparent that 2D cultures have many limitations, including the inability to accurately mimic the in vivo microenvironment of cancer, which in turn affects not only the morphology, drug response, and proliferation rate of the cells [61,62], but as we describe herein, also the genetic profile of the heterogeneous tumor cell populations. Furthermore, established GBM cell lines do not remain clonally or genetically stable during culture, which can lead to discrepancies in drug sensitivity assays [63]. Recent genomic sequencing of the widely used GBM cell lines U87 and U251 revealed that these cell lines had gained numerous mutations owing to decades of 2D cell culture. Furthermore, it is believed that there might be a large number of subclones of U87 cells in labs across the world hampering experimental reproducibility [64]. As such patient-specific models, which are significantly “closer” to the tumor from which they were derived offer incredible potential for cancer research moving forward.

Patient-derived primary cultures of gliomas have been used to eliminate some of the pitfalls of established cell lines. However, many, but not all, patient-derived cells are difficult to maintain in in vitro culture. Often, these cells can undergo senescence or lose subsets of clones due to artificial selection pressures in 2D cell culture. Various research studies have confirmed that patient-derived cell lines undergo ongoing genomic evolution when cultured in a 2D cell culture method, but this was thought to occur over multiple passages and years of culturing on plastic for this genetic drift to occur [65]. The genetic drift occurring in these cells has been appreciated for quite some time now, but our work shows that it takes only minimal exposure of these cells to a traditional cell culture environment to induce these genetic mutations leading to considerable change in response to treatment deviating from the PTCs that were established with the same patient cells immediately following isolation. This is clearly shown visually in our RNAseq analysis in which PTCs, drug-treated PTCs, and originating biospecimens cluster together by the patient, while in contrast, 2D cultures typically cluster together regardless of the patient (Figure 6). These data underscore the critical need to reduce the use of highly artificial conditions for culturing primary cells since once they contact 2D plastic conditions, even only for a short duration, they no longer accurately recapitulate the tissue from which they were derived.

Interestingly, RNAseq analysis also suggests that the PTCs likely supported the growth or maintenance of ECM-modulating cells, cells of neuronal lineages, and immune cell populations, such as myeloid cells, given the greater consistency of myeloid specific gene expression between the tumor tissue and PTC samples (Figure 6). This is not surprising, given our previous efforts to generate immune checkpoint blockade-responsive PTCs by integrating and maintaining patient-matched immune cells within the PTCs [21,22,23,24,30]. Given the success of immunotherapy in several other solid tumors and a growing understanding of the unique immune landscape in the brain in the last few years, there has been a renewed interest in harnessing the immune system as a therapeutic measure against GBM [66]. The microglia, the resident immune cells of the central nervous system, and peripheral macrophages, which are drafted to the brain microenvironment by GBM tumor cells, are the two major players in the immune microenvironment of GBM [67]. The number of microglia/macrophages has been observed to be increased in a high-grade glioma microenvironment compared to low-grade gliomas and has been shown to constitute as much as 30% of the entire tumor biomass. The distinct phenotypes of these cell types associated with GBM are referred to as glioma-associated macrophages and have demonstrated a significant role in disease progression and immune escape [68]. Unfortunately in the case of GBM, clinically pertinent patient-derived disease models faithfully recapitulating the unique immune compartment are lacking, which represents a bottleneck for immunotherapy testing [4]. It is important for glioblastoma models to replicate the unique immune landscape which exhibits both intra and interpatient variability as these immune cells play an important role in how the tumor cells respond to treatment [69]. Since we have shown that our constructs preserve the immune cells present in the tumor and that we can immune-enhance PTCs to be immune-reactive [21,22,30], a natural next step for our team will be to employ these approaches towards GBM. For example, we recently published pilot data suggesting that we can use immune-enhanced PTCs to train or educate naïve T cells from the same patient to recognize tumor antigens from the PTCs [21]. These trained T cells could then be utilized as cellular immunotherapy against normal PTCs that had not been exposed to the T cells. This approach can be viewed as an alternative to CAR T cell therapy that is free from genetic manipulation. To date, we have only employed this approach in the context of melanoma, but it could offer an effective therapeutic option in GBM, as we could train T cells or natural killer cells against constructs containing immune-suppressive elements of the GBM tumor microenvironments.

We should be clear that despite the research community’s work to evolve from the original Verhaak et al. subtyping of GBM into Classical, Proneural, Mesenchymal, and Neural subtypes [45], we did utilize aspects of this subtyping approach to describe heterogeneity in our PTCs while acknowledging the limits this approach has had in influencing clinical outcomes. Today, new classification schemes have been presented, based on characteristics such as stemness or immune signatures [70,71,72]. However, which classification approach will yield significant advances clinically has yet to be determined. This argues for the implementation of patient-specific model systems that can yield personalized empirical treatment response data. Our PTC platform offers this capability.

Despite our PTC model being an effective disease modeling system for GBM that effectively maintains numerous tumor subpopulations and the cellular heterogeneity and genetic profile of the originating tumor, this platform still has limitations. First, in the clinic, the blood–brain barrier (BBB) presents a significant hurdle for delivering treatment systemically to any brain tumor. While the BBB is critical in protecting the brain from external compounds, it also serves as a barrier that most drug compounds cannot normally cross. As such, BBB mass transport properties have resulted in only several drugs being available to GBM patients. The PTC model described here lacks a BBB component, making assessment of chemotherapeutic agent BBB transport efficiencies impossible [73]. However, by utilizing our experience in organ-on-a-chip systems, we are currently working to incorporate BBB components into our system, thus addressing the issues mentioned above. In preliminary work, we have been able to biofabricate a cerebrovascular unit-on-a-chip and have generated data showing that different GBM cell lines have significantly different secretome profiles, which impact BBB integrity differently. We hope that in these ongoing studies, we will soon be able to incorporate GBM PTCs to evaluate how tumor cells impact the BBB, but also how these dynamic interactions influence drug bioavailability to the tumor.

One of the strengths of our platform can also be viewed as a limitation. Specifically, the relative simplicity of our thiolated gelatin and HA hydrogel system is valuable in it is successful at establishing viable PTCs not only from gliomas but numerous other tumors [18,19,20,21,22,23,24]. This simplicity means that the system is defined and could be translated for clinical or clinically linked use more easily than many other biomaterials. Despite its simplicity, the chemistries we work with enable systematic yet effective customization of this hydrogel platform using methacrylate-, acrylate-, thiol-, or maleimide-modified proteins, including collagens, fibronectin, and laminin, thus increasing complexity while maintaining a defined composition. Basement membrane extract biomaterials, such as Matrigel, are still not well-defined, and it is impossible to identify every constituent of such a biomaterial. However, with our current stripped-down ECM system, we do lose some of the potency that biomaterials such as Matrigel possess; although our system does include pendant heparin chains grafted to the HA polysaccharide backbone, enabling immobilization of heparin-binding growth factors. It is likely that due to our simpler system lacking variable endogenous growth factors, we may not be able propagate patient-derived tumor cells to the lengths possible in Matrigel. However, we believe that with simple alterations to the physical microenvironment through additions such as the cell adhesion proteins mentioned above, immense support can be imbued to difficult-to-culture cells. We demonstrated this concept nearly a decade ago in the context of in vitro maintenance of primary human hepatocyte cultures [74].

In conclusion, we have demonstrated that our HA-based hydrogel glioma PTCs can faithfully recapitulate many of the unique microenvironment components of these tumors, along with preservation of not just tumor cell subtypes, but also the non-tumor cell types which play an important role in tumor initiation, progression, and maintenance. To this end, we found that our approach to generating PTCs for GBM is a platform technology that can maintain the genomic profiles of patients’ tumors more effectively than traditional 2D cell culture methods. Thus, we believe this PTC system provides a potent platform with which to perform optimized drug screening assays in a precision medicine context to predict chemotherapeutic responses in patients, which can then be employed to improve clinical strategies in the clinic in a patient-by-patient manner.

## Figures and Tables

**Table 1:** Sample information for glioma biospecimens utilized in this study.

BT #	Biospecimen Weight	Cell Yield	PTCs?	Response to Temozolomide	Response to P53 Activator	Enhancing	Tumor Location	Glioma Diagnosis	Grade	IDH Status	MGMT Methylation	P53 Status
BT4	1000 mg	10 million	P1, P2	No	Yes	y	temporal	Glioblastoma	4	IDHwt	methylated	-
BT7	250 mg	20 million	P0, P1	Yes	Yes to 100 μM	y	frontal paramedian	Glioblastoma	4	IDHwt	indeterminate	-
BT11	1 g	3 million	P0, P1	Yes to 500 μM	Yes to 10 μM	y	parieto-occipital	Glioblastoma	4	IDHwt	absent	-
BT13	10 g	16 million	P0, P1, P2	Yes to 100 μM	yes to 10 μM	y	temporal	Recurrent Glioblastoma	4	IDHwt	absent	-
BT15	800 mg	2 million	P0	No definitive data—No	No definitive data—No	y	frontal	Glioblastoma	4	IDHwt	absent	-
BT1	-	10 million	P0, P1, P2	Yes to 100 μM	NA	n	Parietooccipital	Astrocytoma	3	IDHmu	methylated	positive
BT9	10 g	12 million	P0, P1	Yes to 1 mM	yes to 10 μM	n	frontal	Astrocytoma	3	IDHmu	Not tested	positive
BT10	5 g	7 million	P0, P1	Yes to 1 mM	yes to 10 μM	n	septum pellucidum; frontal, optic tract	Astrocytoma	2	IDHmu	methylated	positive
BT16	-	10 million	P0	No definitive data—No	No definitive data—No	n	frontal	Oligodendroglioma (1p/19 codel)	2	IDHmu	methylated	negative

## Data Availability

The original contributions presented in the study are included in the article/supplementary material, further inquiries can be directed to the corresponding authors.

## Supplementary Materials

The following supporting information can be downloaded at: https://www.mdpi.com/article/10.3390/mi17030276/s1, Figure S1: IHC staining of PTC sections shows the preservation of biomarkers associated with gliomas; Figure S2: Passaging of glioma cells in 2D traditional tissue culture drives shifts in drug responsiveness in 2D cell cultures; Figure S3: P53 pathway activation using the experimental compound NSC59984, stratified by grade, shows efficacy in the majority of glioma PTCs, regardless of grade; Figure S4: Treatment of glioma PTCs with an experimental P53 pathway activating compound NSC59984 has efficacy in most samples; Figure S5: Treatment of A172 constructs with an experimental P53 pathway activating compound NSC59984 increased expression of P21 expression compared to control.
