# Supplementary material for: Hyaluronan-Based Glioblastoma Tumor Constructs Maintain Patient Tumor Drug Responses and Genomic Parity
Source: Micromachines (Basel). 2026 Feb 24;17(3):276. doi: 10.3390/mi17030276 (PMC13028059; doi:10.3390/mi17030276)
Supplement: Supplementary file 1 [file micromachines-17-00276-s001.zip › micromachines-4049631-supplementary.pdf]

Supplementary Materials

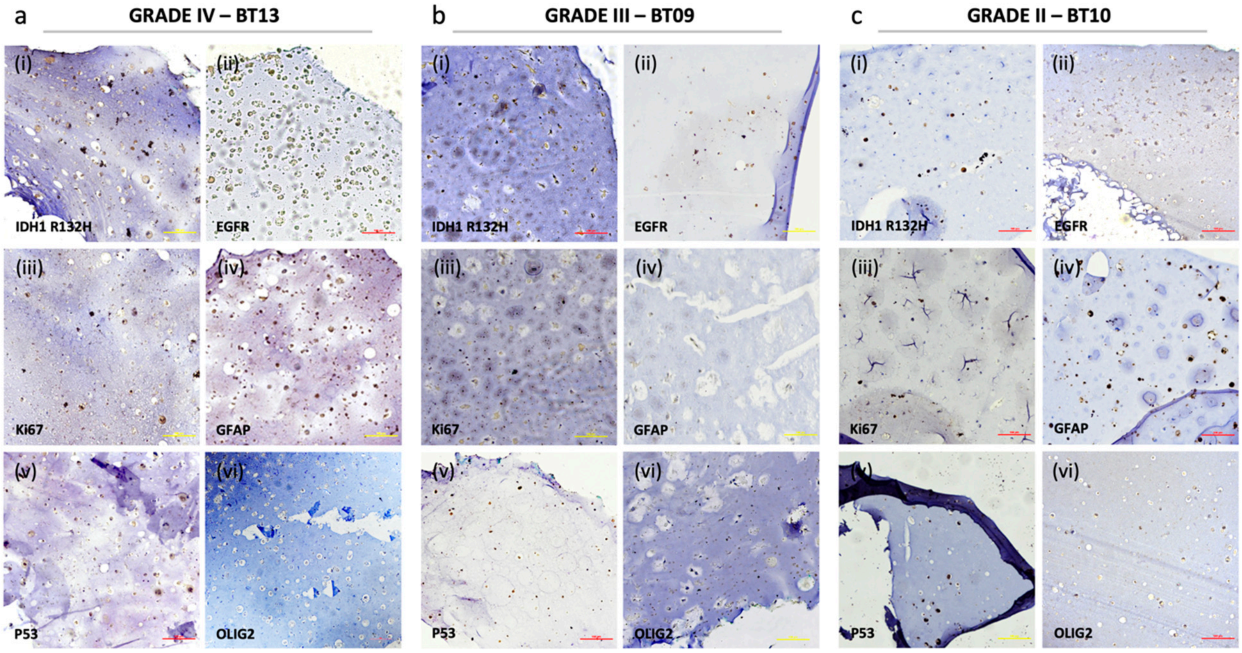

Supplemental Figure S1. IHC staining of PTC sections shows the preservation of biomarkers associated with gliomas. Shown are antibody-stained PTC sections from a) a grade IV, b) a grade III glioma, and c) a grade II glioma. Biomarkers: (i) IDH1 R132H, (ii) EGFR, (iii) Ki67, (iv) GFAP, (v) P53), and (vi) OLIG2. Scale bars: 100 μm.

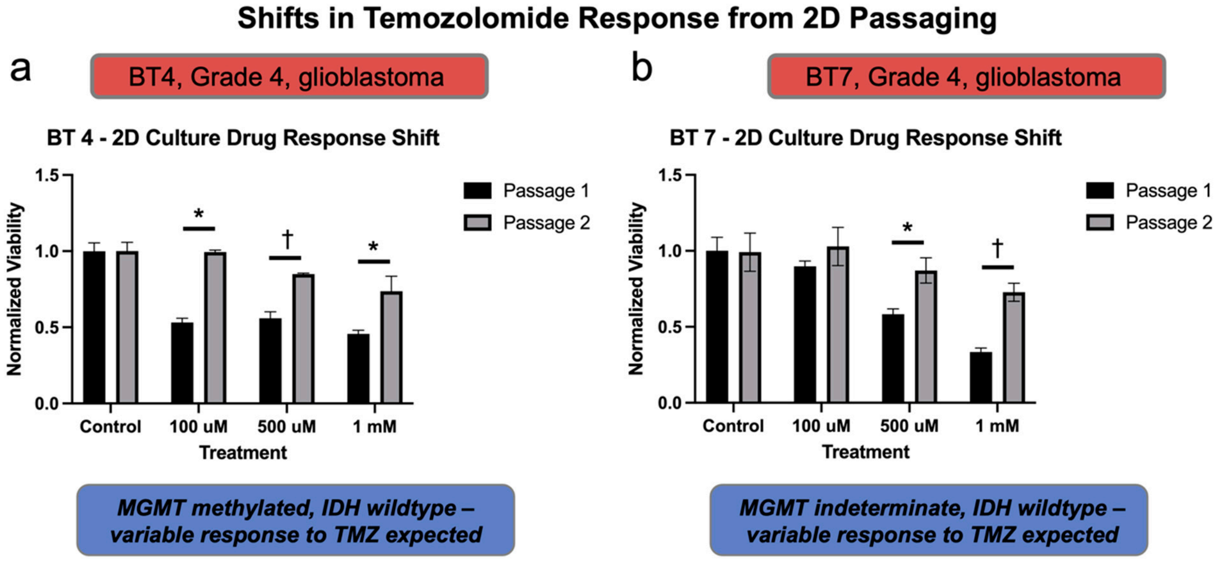

Supplemental Figure S2. Passaging of glioma cells in 2D traditional tissue culture drives shifts in drug responsiveness in 2D cell cultures. a-b) ATP activity quantification showing temozolomide response differences between passages of glioma PTCs BT4 and BT7. Statistical significance: * $p < 0.5$; † $p < 0.01$.

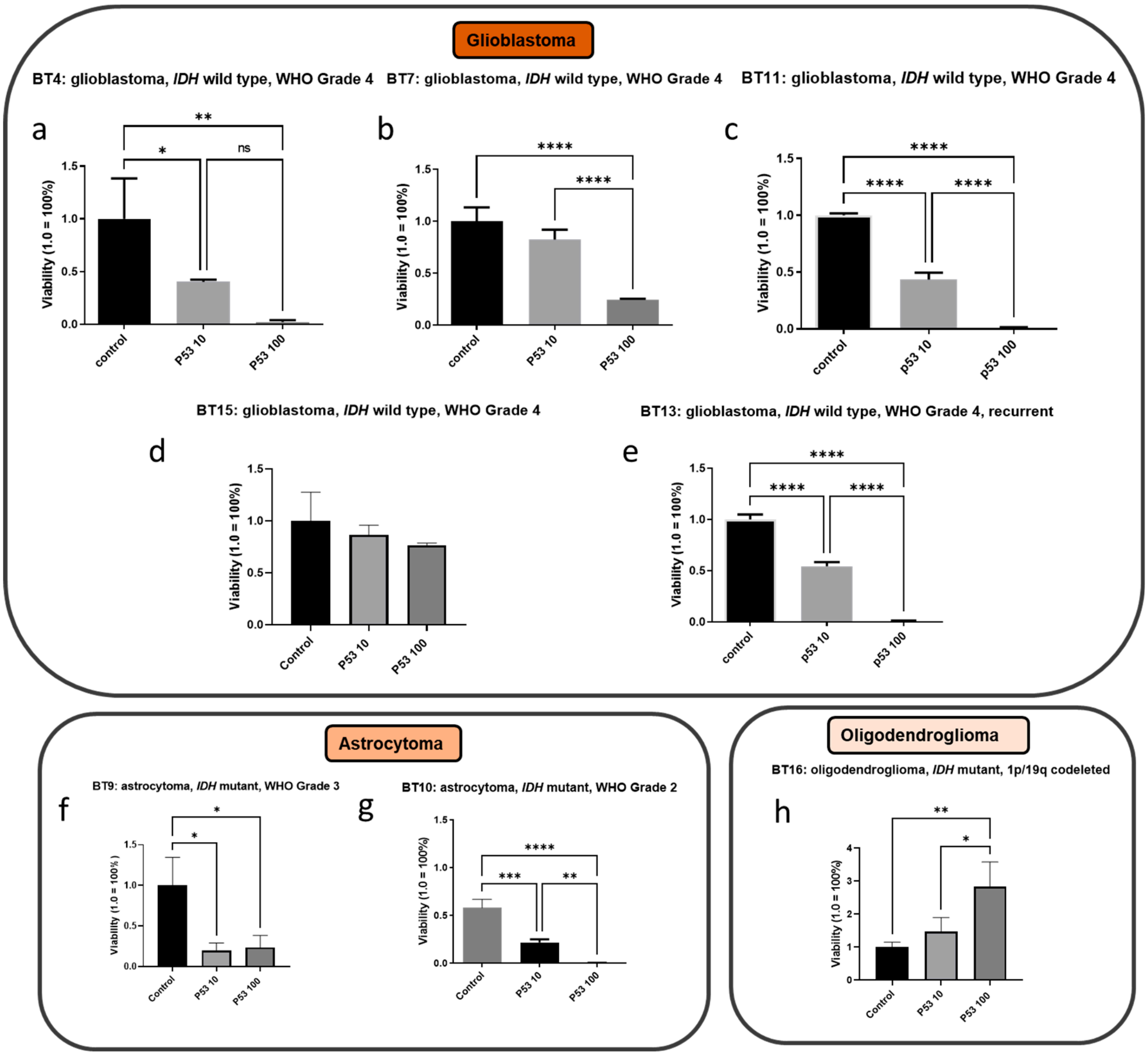

Supplemental Figure S3. P53 pathway activation using the experimental compound NSC59984, stratified by grade, shows efficacy in the majority of glioma PTCs, regardless of grade. ATP activity quantification is proportional to PTC viability. Statistical significance: * $p < 0.05$; ** $p < 0.01$; *** $p < 0.005$; **** $p < 0.001$.

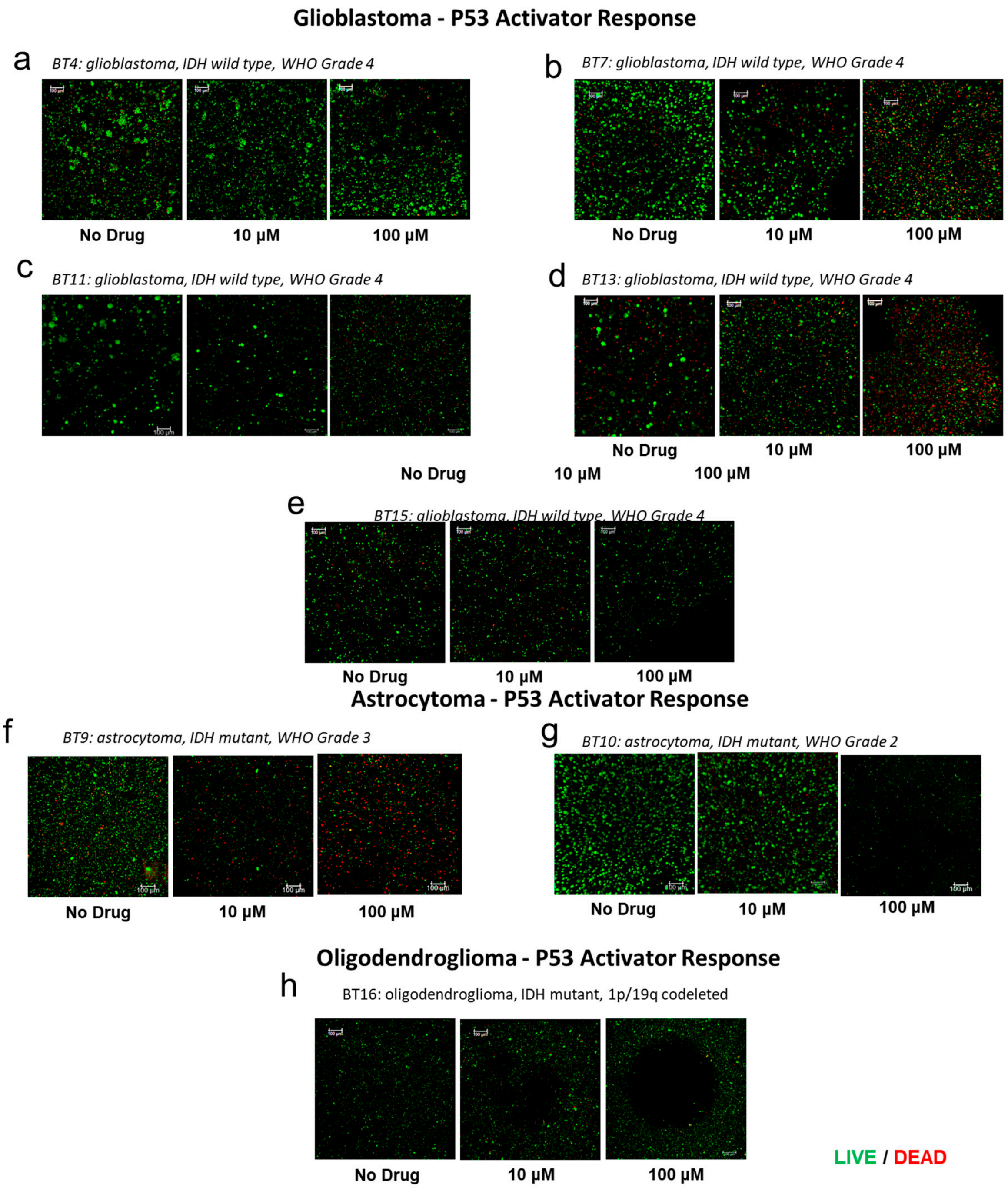

Supplemental Figure S4. Treatment of glioma PTCs with an experimental P53 pathway activating compound NSC59984 has efficacy in most samples. Green fluorescence – calcein AM-stained viable cells; Red fluorescence – ethidium homodimer-1-stained dead cell nuclei.

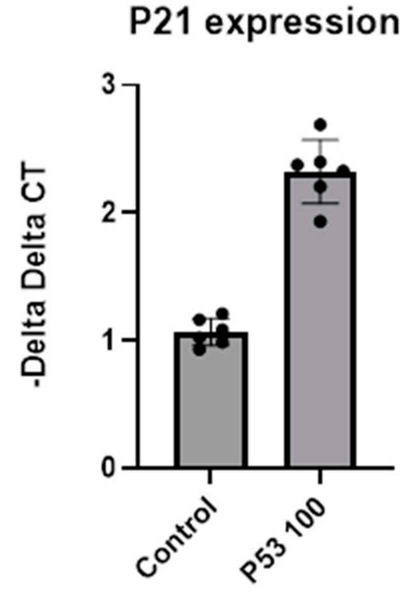

Supplemental Figure S5. Treatment of A172 constructs with an experimental P53 pathway activating compound NSC59984 increased expression of P21 expression compared to control. (−ΔΔCt) in p21 gene expression of A172 constructs when treated with P53 activator NSC59984 (100µM) compared to control A172 constructs.
